# Novel Chemotherapy Modalities for Different Cancers

**DOI:** 10.7759/cureus.45474

**Published:** 2023-09-18

**Authors:** Divya V Lohiya, Ashok M Mehendale, Drishti V Lohiya, Harsh S Lahoti, Vidhi N Agrawal

**Affiliations:** 1 Preventive Medicine, Jawaharlal Nehru Medical College, Datta Meghe Institute of Higher Education and Research, Wardha, IND; 2 Otolaryngology, Jawaharlal Nehru Medical College, Datta Meghe Institute of Higher Education and Research, Wardha, IND

**Keywords:** cyclodextrins, polymeric micelles, cellular absorption, nanosystems, hydrolytic media

## Abstract

Even though many of the approved drugs still have high systemic toxicity due to a lack of tumor selectivity and present pharmacokinetic drawbacks, like low water solubility, that negatively influence the drug circulation time and bioavailability, the anti-cancer study has produced commendable results in recent years. The stability tests carried out under stressful exposure to high temperatures, hydrolytic media, or light sources during their development or under moderate settings have shown the vulnerability of anti-cancer medications to various factors. Because of this, the development of degradation products is considered hospital waste in pharmaceutical formulations and the environment. Until now, various formulations have been created for attaining tissue-specific therapeutic targeting, lowering harmful side effects, and enhancing drug stability. To boost the specificity, efficiency, and durability of active molecules that are targeted in cancer therapy the invention of prodrugs is the potential approach. The latest study illustrates that the solubility, pharmacokinetics, cellular uptake, and stability of chemotherapy drugs can be improved through the incorporation of them into vesicular systems, such as polymeric micelles or cyclodextrins, or via nanocarriers containing chemotherapeutics linked to monoclonal antibodies. In this review article, we provide an overview of the most recent advances in the field of designing very stable prodrugs or nanosystems that are powerful anti-cancer medications and their actions on the body.

## Introduction and background

One of the most frightening illnesses of the 20th century is malignancy; it is still increasing and becoming more common in the 21st century. Every other person has a lifelong risk of developing this malignancy, shocking among all the present affairs [1]. Cancer is a complex series of diseases that develop one after another, accumulating over time to produce a field of cells capable of unregulated growth, and these unregulated cells resist the body's normal mechanisms for cell death. "Karkinoma" is a Greek phrase originating from the term Cancer, depicting projections resembling appendages [2]. Various genetic and epigenetic alterations and chemical carcinogens brought on by repeated exposure to different types of cancer-causing agents such as smoked tobacco, ultraviolet light, persistent injury to tissues of the body, and some virus-causing infections are primarily responsible for the origin, advancement, and growth of this deadly malignant ailment [3-5]. These malignancies' target of wrecking is human tissues that are being enlisted and to little range change into pathological creatures or the elementary unit of the tumors [6]. The term "chemotherapy" is attributed to Nobel prize-winning German physician Paul Ehrlich who researched and studied the application of drugs to cure infectious diseases. He was also one of the first scientists to explore the potential of several drugs for disease prevention using animal experiments. The use of arsenic is said to have started in the early 1900s. Surgery and radiotherapy were the two main methods for treating cancer in the 1960s [7]. The objective of this article is to provide information regarding novel chemotherapeutic agents with respect to cancer.

## Review

Search methodology

The search was done in different databases like Pubmed, Google Scholar, and Scopus using key terms for novel chemotherapy in the treatment of cancer. Related articles over the last 15 years were searched, which included full text, reviews, book articles, website reports, and online published reports. About 78 articles were obtained. After screening for duplicate, suitability, inclusion, and exclusion criteria on the basis of the quality of the article, a total of 38 articles were shortlisted and included in the final review.

Current scenario for the need for chemotherapy

World Health Organization (WHO) made statistical data which bespeaks the immediate necessity for improved cancer restorative choices with better efficacy and fewer side effects [8]. The WHO's statistical data showed a hurried need for enhanced cancer therapeutic options with increased efficiency and few adverse effects [9]. Malignant cells are extremely difficult to treat selectively with traditional chemotherapeutic medicines since they arise from the body's healthy cells [10]. Mechanisms are usually modified by carcinogens and could halt or exaggerate some of the normal human physiological processes [11]. Active chemotherapy treatments have been associated with a significant increase in the toxicities driven on by the body's non-cancerous tissues, particularly those in the skin, spleen, liver, and other important organs. Due to their tendency to only impact tumor cells and their low level of toxicity, the application of chemotherapeutic drugs found in cancer cell-targeted nanoparticles has risen. But for these nanoparticles to use their therapeutic effect on the target cells, they must overcome limitations such as biological barriers and the microenvironment surrounding the tumor [3,12]. One major negative aspect associated with conventional cancer chemotherapy is the possibility of overdose toxicity brought on by cytotoxic drug exposure to the non-tumor cells. To date, molecularly targeted drugs like monoclonal antibodies and specific kinase inhibitors have successfully treated patients by getting around this constraint. Increasing effectiveness using an antibody-drug conjugate (ADC) is the cleverer way and decreases systemic side effects. ADCs use antibodies to deliver a strong cytotoxic chemical to tumor cells in a targeted manner, greatly increasing the therapeutic index of chemotherapeutic drugs [13].

Some newer drugs for different types of cancer treatment

Trastuzumab Deruxtecan (Approved by FDA on August 5, 2022)

Emanate from the evidence that is accessible at the current: A newer-generation ADC that assures almost all the requirements is trastuzumab deruxtecan (DS-8201a) medicament. The hardback freightage in DS-8201a is highly overpowering. It also has a huge drug-to-antibody ratio, is tumor-respective, is homogeneous, has a firm linker-payload in the circulation of the body, splinter, and has a low tectonic half-life cytotoxic agent that may exert an observer effect. DS-8201a may render a favorable cure with outstanding prospects for managing human epidermal growth factor receptor 2 (HER2) intimating malignancies in clinical scenarios according to its presymptomatic features. In a HER2-positive gastric cancer NCI-N87 model, DS-8201a demonstrated a HER2 expression-dependent cell growth-inhibitory effect and promoted tumor regression with a single dosage at more than 1 mg/kg. DS-8201a's anti-HER2 binding and antibody-dependent cellular cytotoxicity (ADCC) activity were equivalent to that of an unconjugated anti-HER2 antibody. DS-8201a exemplified suitable safety outlooks in a phase I trial, potential therapeutic efficacy, and a broad therapeutic index [13].

A subsequent-generation medication named trastuzumab deruxtecan (DS-8201a): ADC relies on the earlier accessible verification that fulfills those requirements. The latest warhead in DS-8201a is inadequate, tumor-specific, cleavable, and has an extremely high drug-to-antibody ratio. In addition, it is a lethal agent with an extremely short in vivo circulatory half-life that has a chance to have a benign effect. DS-8201a may be a victorious treatment with a substantial guarantee for handling HER2-expressing malignant tumors in clinical settings based on its preclinical characteristics. In the phase I trial, DS-8201a exhibited adequate safety profiles, potential productiveness for therapy, and a huge therapeutic index. The breast sufferer with positive HER2 malignancy who had formerly taken treatment demonstrated long-lasting anti-cancer effects with trastuzumab deruxtecan. Interstitial pulmonary disease was seen in a minority of people, along with nausea and myelosuppression, prompting strict scrutiny and awareness of pulmonary symptoms [14].

Treatment of breast cancer with trastuzumab deruxtecan versus trastuzumab emtansine: In collation with trastuzumab emtansine, trastuzumab deruxtecan is linked with a reduced likelihood of disease progression or fatalities among individuals who have positive HER2 cancer of the breast who had formerly received trastuzumab along with a taxane treatment. It was discovered that those using trastuzumab deruxtecan were more vulnerable to developing interstitial lung disease and pneumonitis [15].

Bevacizumab (Approved by FDA on October 11, 2006​​​)

The VEGF-A-selecting monoclonal antibody bevacizumab (Avastin®), which was first sanctioned as an angiogenesis blocker and was also one of the untimely focused pharmaceuticals, was made approachable for clinical usage more than 15 years ago. Bevacizumab, the earliest of a newer class of anti-cancer drugs, remains to give an extensive range of anti-angiogenetic therapy. In addition to cancer of the breast and non-small-cell lung cancer, its indicators now include renal cell carcinoma, cancer of the ovaries, cervix cancer, and glioblastoma. It was initially accepted for treating metastatic colorectal cancer when amalgamed with chemotherapy [16]. More than 15 years prior, the VEGF-A-selected monoclonal antibody (Avastin®), one of the earliest targeted medications, was made accessible for clinical use. It was initially rubber-stamped as an angiogenesis inhibitor. The first of an entirely novel family of anti-cancer medicines, bevacizumab, still pursues to offer the widest range of anti-angiogenic therapy. Its guidance now also includes brain tumors, renal cell carcinoma, cancer of the ovary, cervix cancer, and metastatic cancer of breast and non-small-cell lung cancer. When used with chemotherapy, it was initially authorized to manage colorectal cancer [17].

Mirvetuximab Soravtansine-Gynx (Approved by FDA on November 14, 2022)

The efficacious tubulin-targeting maytansinoid termed DM4 is amalgamed with a folate receptor (FR)-binding antibody to form the mirvetuximab soravtansine (MIRV) combination. MIRV and the investigator's sorted chemotherapy regime were contrasted in the case of plat-resistant epithelial cancer of the ovary (EOC) in a randomized, open-label, FORWARD I investigation of PHASE 3. Compared to chemotherapy, MIRV did not notably improve progression-free survival (PFS) in the sufferers with platinum-resistant EOC. Especially in patients who had elevated FR expression, secondary endpoints typically preferred MIRV. Compared to chemotherapy, MIRV demonstrated a more distinct and feasible safety profile [18].

*Niraparib (Approved by FDA on March 27, 2017​​*​​​​​*)*

Niraparib, presently used to treat ovarian cancer patients, is an oral PARP 1/2 poly (adenosine diphosphate (ADP)-ribose polymerase) drug that has demonstrated therapeutic efficacy. Anyways the existence or lack of gBRCA (breast cancer gene) variations or the position of homologous recombination deficiency (HRD), patients with platinum-sensitive recurrences who received niraparib showed considerably prolonged middle intervals of development-free survival than those who got a dummy medication with relatively moderate bone marrow damage [19]. Niraparib is being used to treat some newly diagnosed advanced ovary neoplasm patients. Despite the existence or the lack of BRCA mutations, it has been established that the drug niraparib has been linked to significantly longer survival without progression among individuals with persistent ovarian cancer after platinum-based treatment. It has been proved that niraparib, which initially suppresses the activity of PARP, substantially improves survival without progression in patients with advanced cancer [20].

Olaparib (Approved by FDA on December 19, 2014)

**Figure 1:**
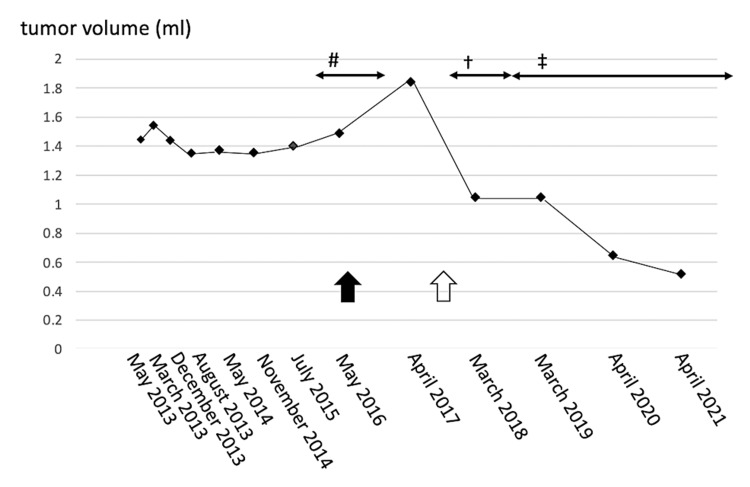
Changes in cerebellopontine angle tumor volume over time and their relationship to therapy for ovarian cancer. The tumor was slowly growing until April 2017, but MRI after the initiation of carboplatin and docetaxel chemotherapy showed a decrease in tumor size, and the tumor continued to shrink during further olaparib chemotherapy. #: carboplatin and paclitaxel; †: carboplatin and docetaxel; ‡: olararib Black arrow: resection of the abdominal tumor; white arrow: ovarian cancer recurrence This image is taken from an open access journal.

Only a fraction of people with metastatic cancer of the pancreas appear to have bequeathed BRCA1 or BRCA2 mutations. Olaparib, a poly adenosine diphosphate-ribose polymerase blocker, has been shown to have anti-cancer properties in this cohort. With continuous olaparib in contrast to placebo, illness-free longevity was increased in those with advancing pancreatic neoplasm and a genetic BRCA characteristic [21]. Olaparib raised the percentage of people with hereditary BRCA1 and BRCA2 mutations (BRCAm) and regional breast cancer (mBC) who survived without recurrence when compared to therapy of the doctor's choice (TPC) in the OlympiAD research study. The expected concluding overall survival statistical research and highlighting of the most severe unfavorable events (AEs) in disposition are demonstrated today to understand the tolerability of olaparib [22]. The relationship between ovarian cancer treatment and cerebellopontine angle tumor size is illustrated in Figure 1 [23].

Rucaparib Tamsylate (Approved by FDA on May 15, 2020)

Rucaparib recently obtained the US FDA's major leap therapy classification for managing growing ovarian cancer among individuals with more than two previous treatments. BRCA modifications, platinum fragility, and cycles of plat-based therapy. Many PARP inhibitors, notably olaparib, and talazoparib, have companion diagnostic (CDx) screening for specific treatment. Their manufacturing is comparable to that of rucaparib. The CDx test, possible PARP inhibitor combination treatment, PARP inhibitor trials in clinical settings, and anticipated resistance mechanisms are all covered in this article. The study covers CDx testing, potential PARP inhibitors combo therapy, clinical trials with medications that inhibit PARP, and expected resistance mechanisms [24].

Nivolumab (Approved by FDA on​​​​​​ March 4, 2022)

In most individuals with gastro-oesophageal adenocarcinoma that fails to show the HER2, standard first-line therapy results in disease progression and deaths within a year [1-4]. According to the randomized, global CheckMate 649 phase 3 study, nivolumab, including chemotherapy, improved complete survival to treatment at 12-month follow-up in patients with gastro-oesophageal, gastric, or oesophageal adenocarcinoma. Through distinct but supportive modes of activity, nivolumab and the CTLA-4 inhibitor ipilimumab promote the development of de novo anti-tumor T-cell responses and the recovery of anti-cancer T-cell activity, respectively. In severely previously treated patients with advanced cancer of gastro-oesophageal junction, treatment with 1 mg kg-1 nivolumab and 3 mg kg-1 ipilimumab indicated considerable anti-tumor effectiveness with an achievable safety profile [25].

Tumor microenvironment (TME): An issue worth monitoring and information to be vigilant about. In the past, radiotherapy and surgery were the only ways to limit tumor growth. Are no longer present from the past. The molecular characteristics associated with malignancies appear to be the cornerstone of any therapy in the complex scenario that is now unfolding. Here, we give an overview of the many cancer treatments available. The vital importance of certain commonly used cancer treatments, including surgery, radiation, chemotherapy, and hormone therapy, is now well-understood in terms of their efficacy mechanisms. The importance of systemic therapy is then highlighted by a summary of the most current and upcoming medications in the era of targeted therapy, including novel antibodies, small compounds, anti-angiogenic, and viral therapy. The identification of novel biomarkers is briefly known. As mentioned, cancer stromal cells actively support the development of malignant cells by secreting paracrine signals. In patients with rectal cancer in clinical stages II to III, neoadjuvant chemoradiotherapy decreased the risk of a localized recurrence. Still, it also postponed the commencement of the best course of action. We compared the outcomes of intermittent versus continuous chemotherapy and radiation treatment when the preoperative drugs fluorouracil, leucovorin, and oxaliplatin (FOLFOX)/bevacizumab were administered [26-28]. Mesenchymal stem cells are integrated, cytokines are generated, growth factor binding increases, and stromal cell dysfunction all contribute to the replenishment and proliferation of cancer cells [29-31].

Impact of TME on tumor density, metastasis, and invasion: These TM traits produce physical barriers that stop medications from entering the body and result in resistance [32].

Targeting the cellular TME using nanoparticles

The nature and functioning of the TME are directly related to the growth and spread of cancers. TME modulation techniques have received a lot of attention recently in cancer immunotherapy. The short medication retention period in TME has hampered the therapeutic effects of immunotherapeutic medicines, notwithstanding their early success. Compared to normal distribution methods, nanoparticles with distinctive physical characteristics and complicated designs can pierce the TME more successfully and convey its crucial constituent. In this work, we briefly discuss the TME's replacements, including dendritic tissue, phagocytes, fibroblasts, tumor vasculature, tumor-draining lymph nodes, and hypoxic conditions, before investigating several nanoparticles that target these components and their potential in the therapy of cancers. In addition to more traditional medical techniques like chemotherapy and radiation, nanoparticles can be used. Because different cancer kinds and people vary, the nanoplatforms' distribution technique might not be effective for all malignancies. The creation of more individualized nanoplatforms will be facilitated by understanding the alterations in TME at different phases of cancer progression [33].

Nanotherepy in cancer diagnosis

Early identification is crucial to the continuous battle against cancer and effective therapy. However, due to the intrinsic limits of conventional cancer diagnostic methods, it is now more difficult to identify cancer early. Nanotechnology has been investigated for detecting extracellular cancer biomarkers and cancer cells and in vivo imaging due to its high sensitivity, specificity, and multiplexed measurement capabilities. The most recent advancements in cancer diagnosis using nanotechnology are summarised in this article. In addition, the difficulties of converting diagnostic techniques based on nanotechnology to clinical applications are explored [34]. Mysterious company in the tumor cancer's microbiome and the microenvironment. Even though it has been established that the intestinal microbiome is a key biomarker and regulator of cancer development and therapeutic response, less is known about the role of the microbiome in cancer in other body sites. Recent research has shown that the local microbiota significantly influences the TME in various cancer types, particularly those that develop in mucosal locations such as the gastrointestinal tract, skin, or lungs [35].

Some newer modalities in cancer treatment

Chimeric Antigen Receptor T cell (CAR-T Cell) Therapy

The latest indented-edge treatment alternative for cancer is the chimeric antigen receptor T cell (CAR-T cell) method of healing therapy. Despite the actuality that treatment with CAR-T cells has exemplified pivotal clinical responses with few subdivisions of B cell leukemia or lymphoma, the therapeutic efficacy of CAR-T cells in hostile solid tumors and hematological malignancies is contrived by several ultimatums. Effective CAR-T cell therapy is hurdled by grave toxicities, which can be lethal, poor anti-tumor venture, antigen bolt, much less trafficking, and minimal tumor assault. In addition, interactions among the host and tumor milieu notably impact CAR-T cell activity. Furthermore, qualified individuals have to expand and execute these therapies. To defeat major obstacles, novel plans, policies, and techniques are to be developed CAR-T cells with increased anti-tumor efficacy and decreased toxicity are needed [36].

Advances in CAR-T Cell Therapy

Whenever put to use in treating hematological tumors, it is more relevant to focus on certain neoplasm varieties; CAR-T cell treatment has emerged with striking advantageous effects inimical to cancer. Merging autologous CAR-T cells presents many drawbacks due to different problematic things like high values, extensive production delays, and limited cell sources. A meticulous advancement that could help to combat the majority of these affairs is the development of a ubiquitous CAR-T (UCAR-T) cell treatment [37].

*Current Progress in CAR-T Cell Therapy*

In doing treatment of various hematological cancers, CAR-T cells have accomplished wonderful clinical victories. Later, the FDA sanctioned two CAR-T cell-based therapeutics, Kymriah (tisagenlecleucel) together with Yescarta (axicabtagene ciloleucel), which is now being used in the US to treat B cell acute lymphoblastic leukemia (B-ALL) and diffuse large B-cell lymphoma (DLBCL), also. Solid malignant tumors remain strenuous to cure with CAR-T cell treatment, despite rectification in treating hematological tumors. Most of the study in this field has been dedicated to enhancing CAR-T cells and minimizing the detrimental impact of the cancer microenvironment on solid tumors. It has a main pivot on better understanding the existing and time to come of CAR-T cell-based treatments for different solid tumors [38].

## Conclusions

The greater incidence, death rates, and recurrence rates for various kinds of malignancy denote that conventional therapy is not enough when done alone or in combination. In a prominent area, there is an opportunity to further assess it. In congruence with reports, the role played by TM in either raising the growth of the cell or hindering the capacity of anti-cancer medications to be effective is among the leading causes of poor chemoprevention. Newer outlooks on dealing with different types of tumors have been studied by the most recent advances in cancer treatment. These changes have led to a greater cognizance of the molecular components that cause neoplasm. Though some of the earlier therapies are still beneficial, they, of course, have certain drawbacks. For instance, although radiation and surgery are advantageous, they only tackle a single concentrated tumor place. Chemotherapy has extremely hazardous side effects but can treat tumors that have spread throughout the body. The defiance of reality may not be the only therapy available today. All of these are still in use and most likely will be used for a time to come, even in the future.
